# Application of the “bubbling” procedure to dead body portraits in forensic identification

**DOI:** 10.1007/s00414-021-02515-0

**Published:** 2021-02-05

**Authors:** Stefan Potente, Frank Ramsthaler, Mattias Kettner, Tomoya Ikeda, Peter Schmidt

**Affiliations:** 1 Department of Legal Medicine, University of Saarland Medical School, Kirrberger Straße, Gebäude 49.1, 66421 Homburg/Saar, Germany; 2 Department of Legal Medicine, Goethe-University Frankfurt Medical School, Kennedyallee 104, 60596 Frankfurt am Main, Germany; 3 Department of Legal Medicine, Osaka City University Medical School, Asahimachi 1-4-3, Abenoku, Osaka, 545-8585 Japan

**Keywords:** Forensic, Identification, Image manipulation, DVI, Unknown body, Perceptual filling-in

## Abstract

**Purpose:**

A procedure is needed for bodies with disfiguring injuries to the face and the use of their portrait for visual identification.

**Method:**

We present the application of a simple image processing procedure, otherwise known as ”bubbling,” which is based on the concept of ”perceptual filling-in,” to images for visual identification in the forensic context. The method is straight forward and can be performed using readily available software and hardware..

**Results:**

The method is demonstrated and examples are shown. The visual recognition of known persons using “bubbled” images was successfully tested.

**Conclusion:**

The “bubbling” procedure for visual identification enhancement is quick and straightforward and may be attempted before escalating to more involved identification methods and procedures.

## Introduction

The practice of visual identification (ID) of dead bodies is performed in forensic medicine under various circumstances, for example, mass death scenarios [1–4]. Despite reports of high error rates of 50% [5], visual ID may be “the only pragmatic option” [6] in some scenarios. In routine autopsy practice, visual ID is performed before autopsy and is generally reliable [7]. In some countries, such visual ID by relatives or any recognizant person is mandatory [8]. The psychological effect on relatives is mixed [9]. Reconstruction, thanatopraxy, or modern embalming techniques may be necessary [10, 11]. Visual ID may be used to identify unknown bodies in morgues [12], and in some countries, the publication of postmortem portraits in different media is a standard procedure [13]. Media publication may also be considered in criminal investigation cases involving unknown bodies, if alternative measures fail for one reason or another. This is however seen as problematic in western cultures, especially in cases with facial trauma. Testing the feasibility of a method called ”bubbling” to correctly recognize and identify a known person after removing trauma stigmata from images was our goal.

## Method

**Fig. 1:**
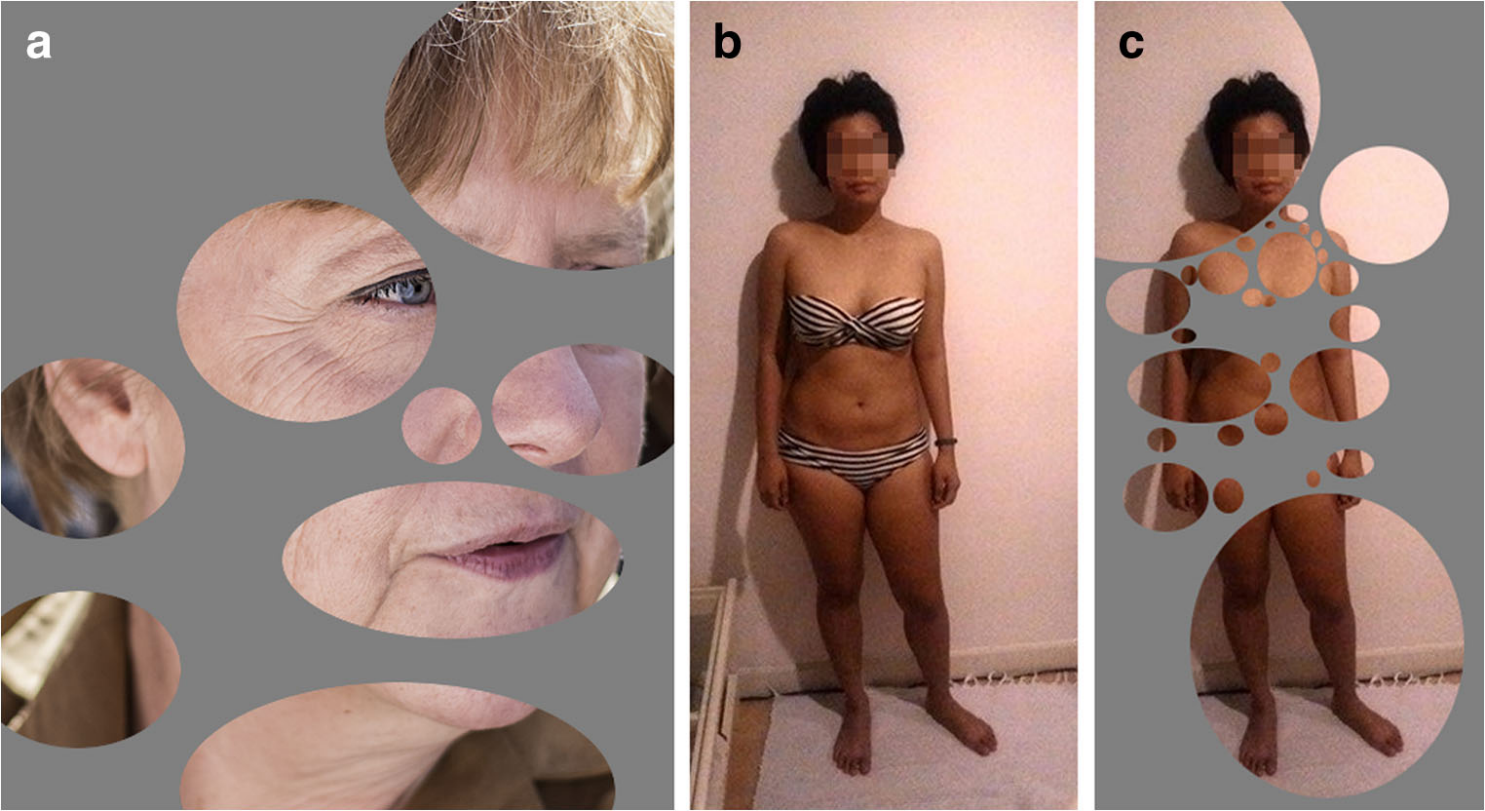
**a** For the recognition of a known face, often only few details are sufficient (German chancellor A. Merkel, 2014, creative commons license CC BY-SA 4.0, author: FNDE). **b**, **c** Typical example of ”bubbling” to imply nudity (author’s friend, pixelation for anonymity only)

**Fig. 2:**
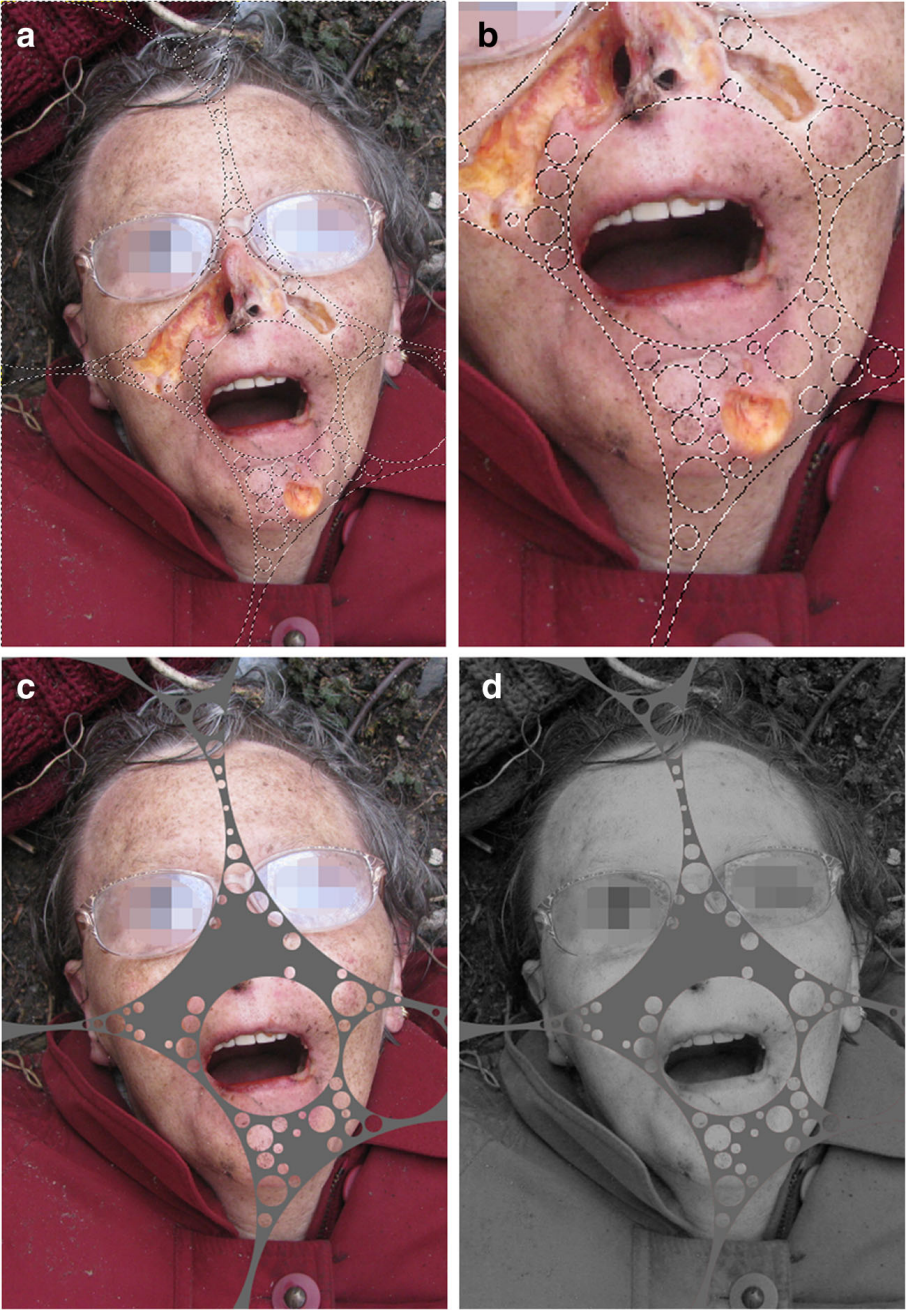
Steps of procedure demonstrated. Natural death, postmortem scavengers (rats). Pixelation for anonymity only. **a** Source picture. **b** Detail of selections. **c** Completed “bubbling.” **d** Additional application of “mono mixer” for selective gray-scaling (GIMP 2.8.16)

**Fig. 3:**
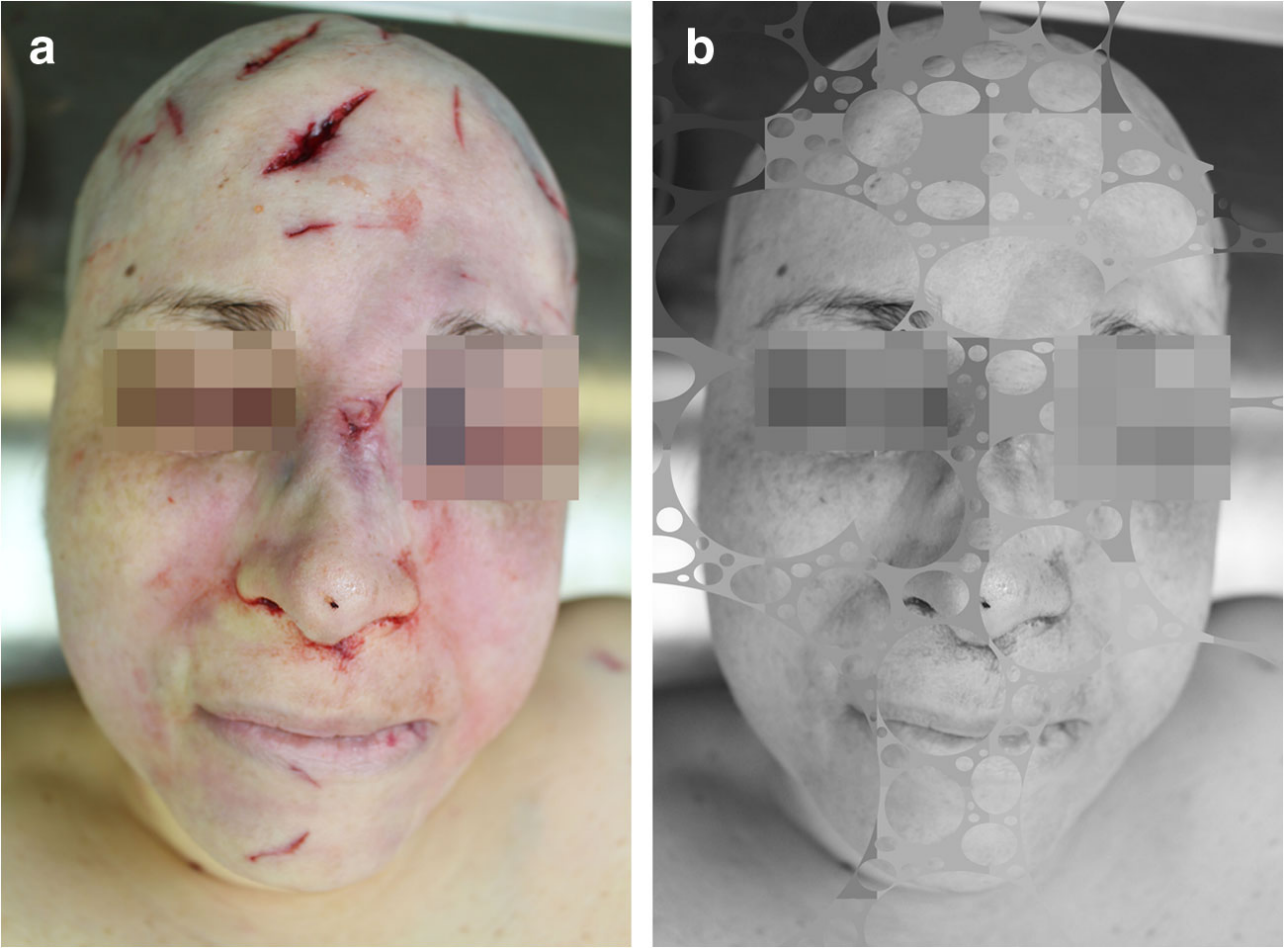
Blows with hatchet, homicide. Pixelation for anonymity only. **a** Before “bubbling.” **b** After “bubbling” and “mono mixer” or selective gray-scaling (GIMP 2.8.16)

Visual recognition is a complex process, which involves a mechanism known as perceptual filling-in, where “a visual attribute [...] is perceived in a region of the visual field even though such an attribute exists only in the surround” [14]. The brain fills in the gaps, based on experience. This principle has been applied unscientifically in online forums to generate proxy-nudity out of harmless source images, in a process dubbed ”bubbling.” Usually in bubbling, harmless images of a target person wearing swimwear or other light clothing is used as the source. Desired content (skin) is selected while undesired content (clothing) in non-selected residual spaces is faded out to create proxy-nudity. The use of round or ellipsoid selections (”bubbles”) seems to promote this process by creating an unobtrusive mesh of organically shaped residual spaces (see Fig. 1, for example). The technical application of the bubbling procedure is straightforward, including the application for facial recognition. A basic image manipulation program is needed (Photoshop®, Paintshop®, GIMP). Then, round- or ellipsoid-shaped selections (”bubbles”) are drawn around undisturbed areas, while undesired (in our case traumatized) space is left unselected. At first, as large as possible bubbles are selected, followed by smaller bubbles in between selections until most of the desired content is selected. Now, this selection is inverted and the residual space in between bubbles is painted in or otherwise obscured, for example using large-scale pixelation. Original color should be retained, since skin tones and hair color may contribute to the identification. De-saturation/gray scaling of bubble content may however be necessary for prominent lividity, bruising, decomposition, or other distracting discolorations (see Fig. 2 for an overview of the process (using GIMP) and Fig. 3 for a result example).

## Results

The general recognition of “bubbled” portraits was tested in an experimental setting. Two student groups from Homburg (*N*=38) and Budapest (*N*=15) were presented with 10 bubbled portraits of national and international public figures in quick succession. For each, the students had to either note the name of the person or mark it “not recognized.” After all bubbled pictures were shown, the unaltered original images were shown and the students could note “I do not know this person” if in fact they did not.^1^ Different outcomes had to be considered:

1. A known person was recognized correctly (“It was A, I knew it!”).
2. A known person was mistaken for another known person (“I thought it was A, but now I see it is B.”).
3. A known person was not recognized (“I did not see that it was A.”).
4. An unknown person was not recognized (“Never seen, no idea!”).
5. An unknown person was wrongly identified as another person (“Not B? Then I don’t know!”).

The total count of queries was 530 (53 × 10). The public figure was confirmed to be known by the student in 382 cases (72.1%). Out of those, 255 queries (66.8%) were recognized correctly, 33 (8.6%) were mistaken for another celebrity, and 94 (24.6%) were not identified.

## Discussion

Many portraits of cases with disturbing, yet circumscribed facial trauma can be treated efficiently and effectively for possible publication by unobtrusively fading out findings. Depending on the individual case, recognition rates are potentially high, as suggested by the outcome of our experiment. In violent mass death such as terror attacks and train accidents, the method may assist in the identification of victims (and perpetrators), especially when for one reason or the other sufficient other means of identification are unavailable. The method may be applied to electronic images quickly and easily, using readily available software and hardware. As far as can be said following our experiment, the method works best with limited, circumscribed findings such as gunshot wounds, cuts, and lacerations. It is advisable to salvage presentable fractions of features whenever possible to retain features such as shape, position, and size for eyes, nose, ears, hairline and others, even if a larger extent of said features is distorted. Use of pre-autopsy, pre-cleaning pictures might sometimes be beneficial for identification (noticeable head wear, make up, jewelry, and clothing). Bloodstains or damage on objects may be included into the ”bubbling” process. Generalized findings without “gaps to fill,” such as swelling, bloating, and discoloration as well as grotesque deformations cannot be improved by the procedure. As to how much visual information may be omitted in the grid depends both on the case and the viewer, so no general rules can be given. However, if large portions of the face must be left blank when ”bubbling,” alternative methods such as forensic artwork, sophisticated image processing, or facial reconstruction techniques may be more suitable.

## Conclusion

The “bubbling” procedure is a fast, inexpensive identification enhancement technique which may be attempted before escalating to more involved procedures.

## Footnotes

1 Celebrities included Lothar Matthäus (soccer player/ coach), Ursula von der Leyen (politician), Heidi Klum (model), Viktor Orbán (politician), Bruce Lee (actor), Greta Thunberg (political activist), Pope Francis, Tom Cruise (actor), Rihanna (singer), Frauke Petry (politician), and Inka Bause (TV presenter). In addition, one person known as a tutor was included as well as one person who was not a public figure and completely unknown to the students (as a control).
